# Autogenous Demineralized Dentin Graft–Based Strategies With or Without I‐PRF and Metronidazole for Ridge Preservation in Infected Sockets: A Randomized Clinical Trial

**DOI:** 10.1111/jre.70092

**Published:** 2026-02-20

**Authors:** Mohamed Talaat Elbehwashy, Manal Mohamed Hosny, Ahmed Elfana, Heba Ahmed Saleh, Karim Fawzy El‐Sayed

**Affiliations:** ^1^ Oral Medicine and Periodontology Department Faculty of Dentistry, Cairo University Cairo Egypt; ^2^ Periodontology Department NewGiza University Cairo Egypt; ^3^ Department of Prosthodontics School of Dentistry, Indiana University Indiana USA; ^4^ Oral and Maxillofacial Pathology Department Faculty of Dentistry, Cairo University Cairo Egypt; ^5^ Clinic for Conservative Dentistry and Periodontology School of Dental Medicine, Christian Albrechts University Kiel Germany

**Keywords:** alveolar ridge preservation, dentin, extraction, graft, implants, metronidazole, platelet‐rich fibrin

## Abstract

**Aim:**

This pilot randomized controlled trial evaluated the efficacy of demineralized dentin graft (DDG) combined with injectable platelet‐rich fibrin (I‐PRF) and metronidazole (MTZ) versus DDG with I‐PRF and DDG alone in alveolar ridge preservation (ARP) of chronically infected extraction sockets.

**Methods:**

Patients with non‐restorable teeth and chronic periapical infection in the maxillary anterior and premolar regions were randomly assigned to three groups (*n* = 18/group): DDG alone, DDG + I‐PRF, and DDG + I‐PRF/MTZ. After extraction, graft materials were placed into sockets. Cone‐beam CT scans at baseline and 6 months assessed vertical and horizontal ridge dimensions. Virtual implant planning evaluated the need for further augmentation. Pain was recorded daily for 7 days using a visual analogue scale (VAS). Bone biopsies at 6 months were harvested during implant placement for histomorphometric analysis.

**Results:**

Fifty‐four patients were analyzed. No significant differences were found in vertical and horizontal ridge dimensional changes (*p* > 0.05). Minimal additional augmentation was needed. The DDG + I‐PRF/MTZ group showed significantly the highest mineralized bone percentage (55.02% ± 3.65%), followed by DDG + I‐PRF (41.14% ± 7.00%) and DDG alone (30.21% ± 3.72%) (*p* < 0.05). Soft tissue proportion histologically was significantly lowest in the DDG + I‐PRF/MTZ group (*p* < 0.05).

**Conclusion:**

All protocols were effective for ARP. Addition of I‐PRF with MTZ enhanced bone mineralization.

**Trial Registration:**

www.clinicaltrials.gov: NCT06374784

## Clinical Relevance

**Background:**

ARP in extraction sockets for subsequent implant placement with periapical pathological lesions remains a clinical challenge due to compromised healing and increased risk of ridge resorption.

**Added Value of This Study:**

This pilot randomized clinical trial demonstrates that combining demineralized dentin graft with injectable platelet‐rich fibrin and metronidazole (DDG + I‐PRF/MTZ) significantly enhances mineralized bone formation and reduces soft tissue infiltration compared to DDG alone or with I‐PRF.

**Clinical Implications:**

Within the limitations of not including a natural‐healing control group, all three DDG‐based strategies supported comparable ridge preservation outcomes in infected sockets. The observed differences in histologic composition may guide clinicians in tailoring ARP protocols when managing sites compromised by apical pathology. However, conclusions regarding superiority over spontaneous healing cannot be inferred from this study, and results should be interpreted with appropriate caution.

## Graphical abstract

This randomized controlled trial evaluated the efficacy of demineralized dentin graft (DDG) combined with injectable platelet‐rich fibrin (I‐PRF) and metronidazole (MTZ) versus DDG with or without I‐PRF in alveolar ridge preservation (ARP) of infected extraction sockets.

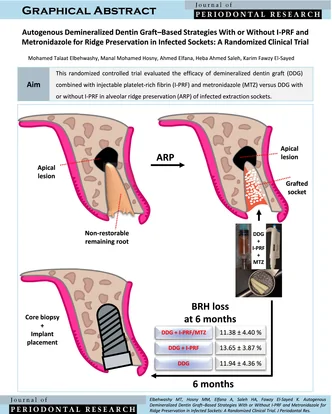

## 1 Introduction

Tooth extraction is usually accompanied by a remarkable reduction in alveolar ridge volume, especially in the anterior maxillary esthetic zone [1] with its very thin buccal bone plates, consisting mainly of tooth‐dependent bundle bone that quickly resorbs following tooth extraction [2, 3]. These dimensional changes can increase the probability of facing a more complex subsequent rehabilitation situation, including the potential need for additional grafting, staged implant placement, or prosthetic compromises [4, 5, 6, 7]. Hence, alveolar ridge preservation (ARP) techniques have been advocated to mitigate these biological alterations, employing various grafting materials to effectively reduce the need for additional bone augmentation procedures at the time of implant placement [8, 9, 10, 11].

Autologous dentin is considered a promising alternative grafting material due to its resemblance in structure, growth factors, and chemical composition to native bone [12, 13, 14], demonstrating effectiveness in ARP [15, 16, 17]. Demineralization of the dentin graft has been further proposed to expose its collagen matrix, liberate growth factors, and thereby enhance its osteoconductive capacity [18, 19]. Recent systematic reviews have reported that autogenous dentin grafts demonstrate favorable outcomes for alveolar ridge preservation, including effective volume maintenance, encouraging histologic and histomorphometric results, and a low incidence of complications. In particular, the demineralized form appears to be a promising clinical alternative for reconstructing alveolar ridge dimensions [20, 21, 22].

The introduction of the low‐speed centrifugation concepts (LSCC) to PRF preparation protocols resulted in a higher sustained release of growth factors [23]. In this context, the liquid injectable‐PRF (I‐PRF), prepared through the LSCC, provides an extended working time before its gelation, allowing for the incorporation and sustained local delivery of various biological agents, including antibiotics [24, 25, 26, 27, 28, 29, 30, 31, 32]. The antibacterial effect of I‐PRF loaded with antibiotics could reduce the post‐operative infection that may happen as a complication after extraction of teeth and lower the need for systemic antibiotic regimens [28, 33]. Metronidazole (MTZ), a synthetic nitroimidazole antibiotic, is the drug of choice in the treatment of chronic dental infections, like abscesses and endodontic pathosis, being effective against anaerobic pathogens [34, 35, 36, 37, 38]. An incorporation of MTZ in PRF demonstrated significant antibacterial effectivity [27] and a reduction in defect depth and volume of intrabony periodontal defects [39]. Additionally, a combination of platelet concentrates with the dentin grafts could enrich it with a variety of growth/differentiation factors and leucocytes, providing further a sticky consistency and ease of handling [40, 41].

Any type of infection may shift socket healing toward a pro‐inflammatory, fibrotic trajectory that can manifest clinically as delayed or erratic healing [42]. In certain cases, like endodontic or periodontal infections, connective tissue infiltration may occur in place of bone deposition, leading to irregular or compromised healing of the extraction site [42, 43]. Thus, the current trial was conducted to assess, for the first time, the effects of ARP in chronic infected sockets, using autogenous demineralized dentin graft (DDG) with metronidazole‐loaded injectable PRF (DDG + I‐PRF/MTZ) versus autogenous demineralized dentin graft with injectable PRF (DDG + I‐PRF), or autogenous demineralized dentin (DDG) alone on alveolar buccal ridge height change (BRH, primary outcome) as well as on changes in palatal ridge height (PRH), alveolar ridge width (RW), pain scores, the need for additional grafting at 6 months and the area percentage of newly formed bone, soft tissue and residual graft in human biopsies histologically (all secondary outcomes).

## 2 Methods

### 2.1 Study Design and Registration

The present study was designed as a pilot randomized, controlled, parallel three‐arm clinical trial with a 1:1:1 allocation ratio to compare DDG + I‐PRF/MTZ‐group (test‐1) to DDG + I‐PRF‐group (test‐2) and DDG‐group (control) in ARP of post‐extraction sockets with chronic infected lesions in the esthetic zone. The study was carried out in compliance with the EQUATOR guidelines and the revised version of the Helsinki Declaration in Finland, 2024. Research protocol, informed consent templates, and biological sample collection requests were approved by the Research Ethics Committee, Faculty of Dentistry, Cairo University, in September 2022 (IRB number: 22|09|10). The research protocol was registered, conducted, and reported in compliance with the CONSORT guidelines.

### 2.2 Recruitment of Participants

Potential candidates requiring dental implant rehabilitation in a single anterior or premolar tooth with periapical chronic lesions [44] in the maxillary esthetic area with periodontally healthy adjacent teeth were screened for eligibility and voluntarily recruited. Teeth were extracted if they were non‐restorable due to tooth fracture, trauma, periapical infection, endodontic complications, and extensive caries. Patients had to be systemically healthy, at least 18 years in age or older, with FMPS of < 15% and an FMBS < 10% [45]. Following extraction, sockets were checked to ensure that they were having a type I, infected socket with intact four walls [43, 46]. Smokers, patients reporting any systemic conditions that may compromise healing or bone metabolism (e.g., uncontrolled diabetes, Paget disease, or hyperthyroidism), having a history of radiotherapy, chemotherapy, or bisphosphonate therapy, and females who were pregnant or lactating were excluded.

### 2.3 Sample Size

The sample size calculation was performed using G*Power version 3.1.9.4 (University of Düsseldorf, Germany) via the F‐test (ANOVA: Fixed effects, omnibus, one way) based on the change in buccal ridge height (primary outcome). Using the mean difference of 0.72 mm and an SD of 0.27 from a previous clinical trial [17] in which similar outcome measurements were performed. An effect size (Cohen's *d*) of 0.48 was calculated, and considering a power *β* = 0.8 and *α* = 0.05, 15 patients per group were required to reject the null hypothesis. The number was increased to 18 patients per group to account for a possible 15% dropout rate, with a total of 54 patients to be recruited for the trial.

### 2.4 Randomization

Extraction sockets were randomly assigned to undergo ARP using either DDG + I‐PRF/MTZ (test‐1), or DDG + I‐PRF (test‐2), or DDG (control) with a 1:1:1 allocation ratio. Sequence generation and concealment were carried out by a single investigator (MH) using www.random.org. Allocation was concealed in serially numbered, identical, and opaque sealed envelopes. KFE was responsible for assigning the allocation of participants into the corresponding study group. All participants were enrolled and equally prepared for the surgical procedure by a single investigator (MTE). After atraumatic extraction, demineralization, and grinding, the intervention allocation was revealed (KFE) to the investigator (MTE) according to the sequence generated. Simple randomization was done for cases from each group eligible for harvesting of bone biopsy before implant placement.

### 2.5 Blinding

Blinding was feasible for the outcome assessors and the statistician. However, it was not possible for the principal investigator to be blinded, given that the surgical procedures of the three groups were basically distinct. Blinding of participants was further not possible, as the participants of the test groups were administered a distinct intervention, requiring venous blood withdrawal for the I‐PRF preparation.

### 2.6 Preoperative Phase

After participants were enrolled in the study, their medical history was recorded, the target teeth were examined clinically, and periapical radiographs were taken (Figure Table S1). Each participant received a thorough explanation of the study's purpose, potential benefits, surgical procedures, possible risks, and timeline before providing written informed consent. Professional mechanical plaque removal was carried out, and oral hygiene instructions were clearly explained and strongly emphasized.

### 2.7 Surgical Procedures

Two weeks later, the tooth extraction visit was scheduled. Following rinsing with 0.12% chlorhexidine mouthwash (Hexitol, ADCO Pharma Co, Cairo, Egypt), 4% articaine HCl with 1:100 000 epinephrine (Artinibsa, Inibsa Dental, Madrid, Spain) was administered. Flapless atraumatic extraction was initiated by performing a supracrestal fiberotomy using 15c surgical blades (KLS Martin GmbH, Tuttlingen, Germany). Following tooth loosening via periotomes (Kohler Medizintechnik, Stockach, Germany) [47] and straight luxators (Zoll Dental, Niles, IL, USA), forceps (Nordent Manufacturing Inc., IL, USA) were used to extract it from its socket, followed by inspection of the integrity of the extraction socket using an UNC‐15 periodontal probe (KLS Martin GmbH, Tuttlingen, Germany). Meticulous debridement and surgical curettage of the extraction socket were done using a bone curette (Helmut, Zepf Medizintechnik, Seitingen‐Oberflacht, Germany) until it was ensured that the socket was clearly free from inflammatory tissue, and the socket was irrigated with saline [48].

The extracted tooth was thoroughly cleaned of any residual periodontal ligaments, cementum, soft tissue attachments, caries, or restorations using a high‐speed diamond stone and irrigation with saline. A longitudinal groove along the root length using a fine tapered diamond stone was made to make sure that every part of the root canal filling materials or pulp tissues was completely removed using 6× magnification loupes (Dr Scope, Minam Optics Co. Ltd., Daegu, South Korea), having a root composed of only dentin from out and inside.

Subsequently, the tooth was ground using a bone rongeur and a hand bone mill (Helmut, Zepf Medizintechnik, Seitingen‐Oberflacht, Germany) to obtain dentin particles with an average size of 1 mm [49]. The dentin particles were then demineralized by immersing them in 0.6 N hydrochloric acid for 30 min [17], followed by twice three‐minute rinsing with phosphate‐buffered saline (dentin washer) and drying using a sterile gauze [16, 50, 51, 52, 53, 54].

Following demineralization, the intervention allocation was revealed, and the graft material was prepared according to the allocation sequence into either:

#### 2.7.1 DDG ± I‐PRF/MTZ‐Group

Ten millimeters of venous blood was collected in a 10 mL sterile vacutainer plastic tube. The blood was centrifuged at 700 rpm (RCF‐max = 60 g) for 3 min with a centrifugation device (45° rotor angulation and a maximum radius of 10.6 cm, Digital Tabletop Centrifuge, Velab, VE‐4000, TX, USA), and then 3 mL of I‐PRF liquid layer was collected from the top of the tube [55, 56, 57]. Three hundred microliters of MTZ (Amrizole 500 mg/100 mL, Amriah Pharmaceutical Industries, Alexandria, Egypt) was withdrawn and added to the I‐PRF and stirred [27, 30]. The I‐PRF loaded with MTZ was then mixed with the demineralized dentin graft particles [12, 15]. The mixture was then allowed to set for 10 min to produce the sticky MTZ loaded demineralized dentin graft (DDG + I‐PRF/MTZ).

#### 2.7.2 DDG ± I‐PRF‐Group

I‐PRF was prepared as previously described and mixed with the demineralized dentin graft producing the sticky demineralized dentin graft (DDG + I‐PRF).

#### 2.7.3 DDG‐Group

Applying only demineralized dentin graft particles (DDG).

The extraction socket in each group was filled with the corresponding graft material, and a non‐crosslinked bioabsorbable collagen membrane (Hypro‐Sorb, Bioimplon GmbH, Gießen, Germany) was used for socket sealing. The membrane was placed in the socket orifice by tucking it buccally and palatally beneath the soft tissue [17] and secured using a hidden‐x internal horizontal mattress 5–0 monofilament polypropylene suture (ASSUT, Pully‐Lausanne, Switzerland) [58] (Figure 1).

**FIGURE 1:**
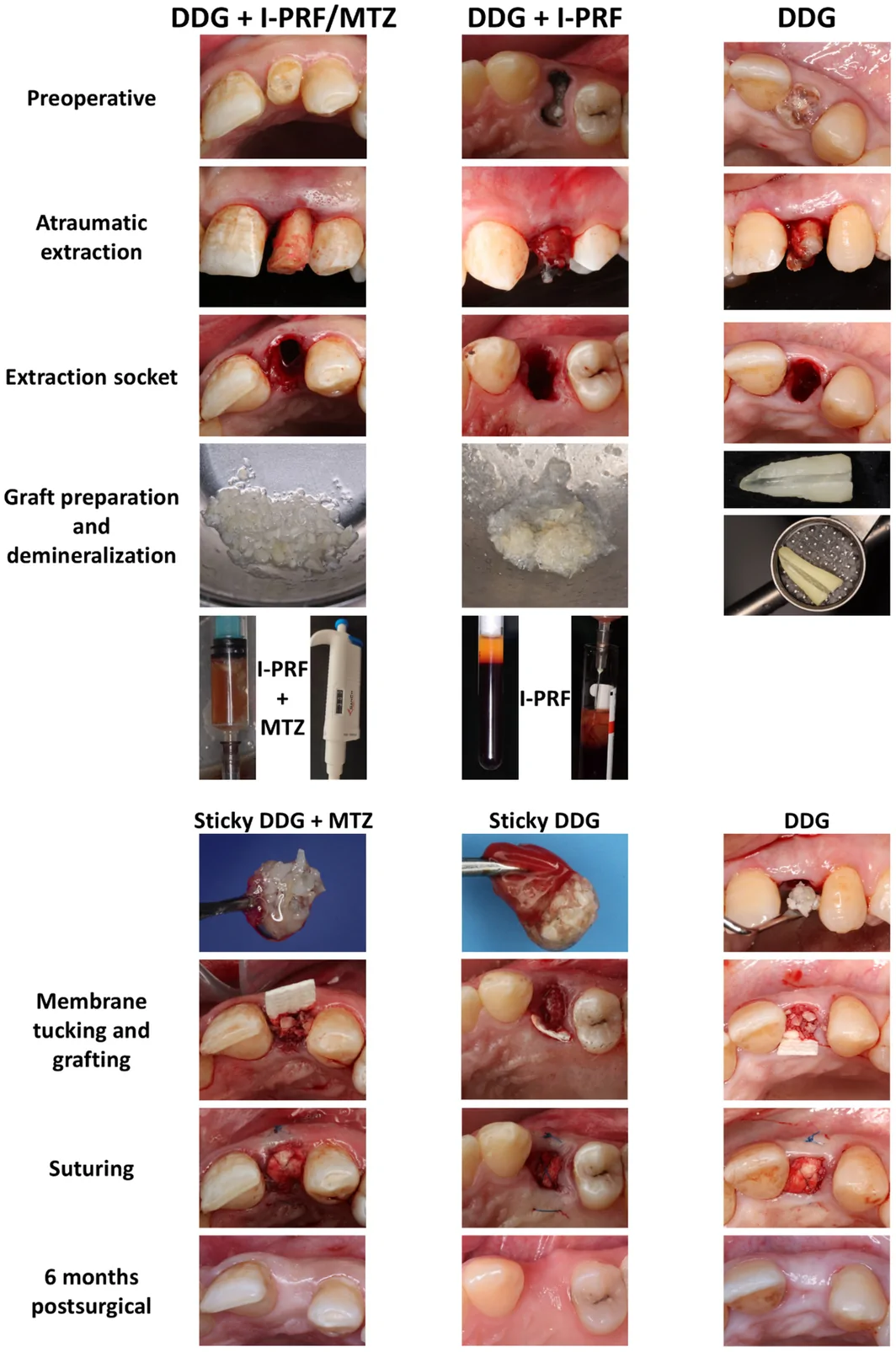
Clinical steps of graft harvesting, preparation, demineralization, insertion, and 6 months of follow‐up in the three groups in three representative patients. I‐PRF withdrawal, mixture with MTZ and DDG in the Test‐1 group, and mixture with DDG in the Test‐2 group.

### 2.8 Post‐ Operative Care and Follow‐Up

Participants were instructed to avoid any trauma at the operative site and prevent any interference with the suture [45]. It was recommended to avoid brushing of the surgical area and resume gentle brushing at the area of the operation after a period of 2 weeks. Ibuprofen 600 mg (Brufen, Kahira Pharmaceuticals, Egypt) was prescribed only to alleviate severe pain, if it happened. A 0.12% chlorhexidine mouthwash was recommended for gentle rinsing, to be used twice daily for a period of 2 weeks [17, 59]. Baseline cone‐beam computed tomography (CBCT) was performed on the same day of extraction. The sutures were removed 2 weeks after surgery, and the temporary PMMA crown bonded with resin to adjacent teeth or integrated within the Essix retainer, not resting on the healing site, was delivered. Final follow‐up visits and CBCT scans were carried out 6 months postoperatively.

### 2.9 Outcomes

#### 2.9.1 Radiographic Outcomes

At parameters of 0.2 mm voxel size, 90 kV, and 8 mA, CBCT scans (Planmeca ProMax 3D Classic, Helsinki, Finland) were obtained at baseline and 6 months post‐operatively. Scans were exported in DICOM format to the 3D viewer software (Ondemand 3D communicator, Cybermed, 1.0.9, Seoul, South Korea).

To evaluate the outcome assessor reliability, buccal ridge height was measured twice with 1 week interval on a sample of 10 CBCT scans not included in the study. The intraclass correlation coefficient (ICC) for single measures, based on a two‐way mixed‐effects model with consistency agreement, was 0.96 (95% CI: 0.86–0.99), indicating excellent intra‐rater reliability.

In baseline CBCT scans, alveolar socket volume [60] and the volume of the periapical lesion were measured in cubic millimeters, as well as the buccal plate thickness at 1 mm apical to the crest [61, 62] (Figure 2). Buccal bone thickness was categorized as either thin or thick based on a clinically meaningful threshold, defined as ≤ 1 mm for thin plates and > 1 mm for thick plates [2]. The volume was estimated using the Cavalieri principle. The surface area of each section or slice was measured, and the sum of the areas was used to estimate the volume using the following formula:

V=t×∑A

where *t* is the slice thickness (including intervals) and ∑*A* is the total sectional area of the consecutive sections [63].

**FIGURE 2:**
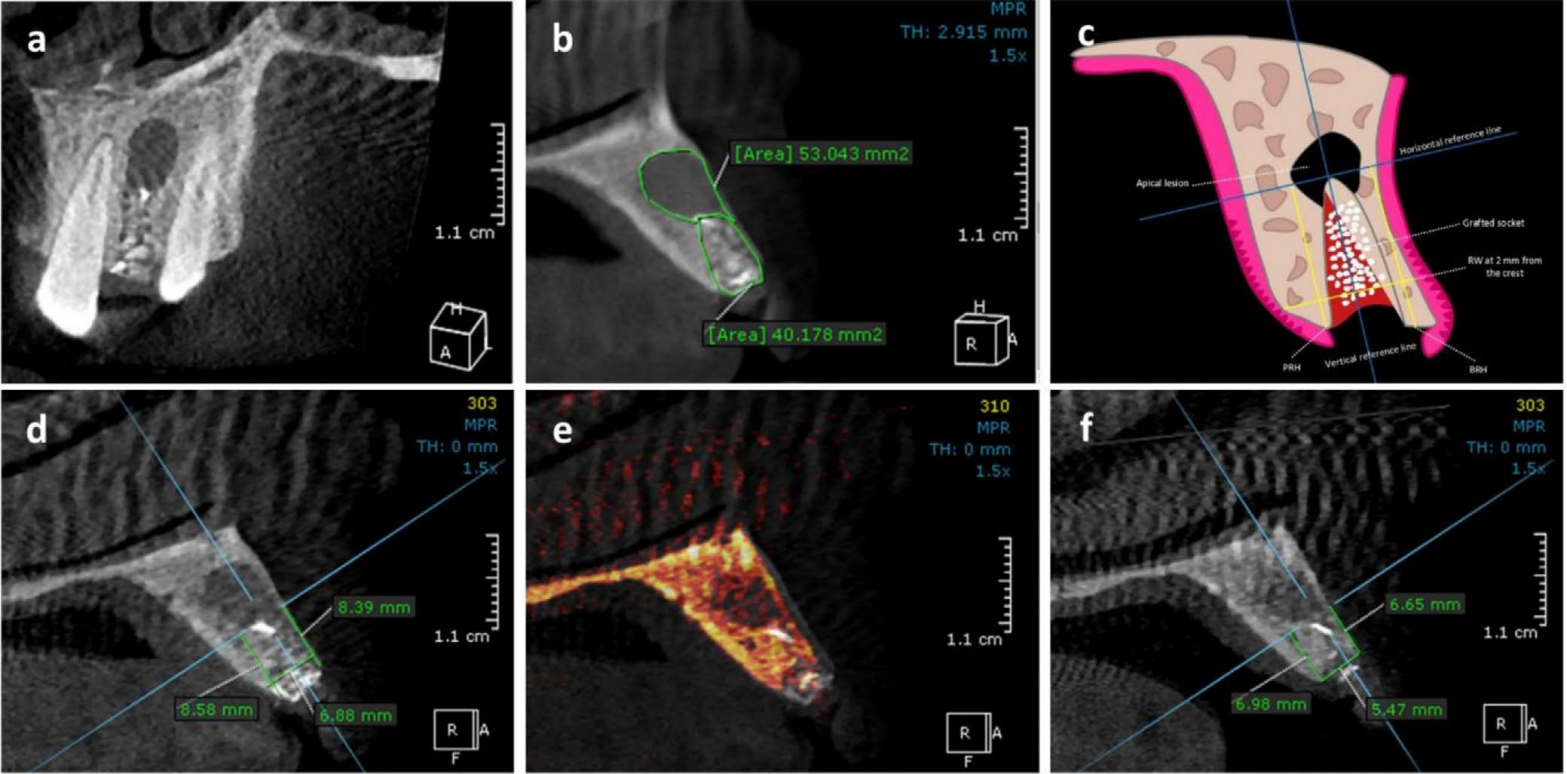
Radiographic examination. (a) coronal section showing the grafted section and the apical radiolucency, (b) measuring the areas of the socket and the apical lesion and slice thickness (TH‐blue) for each slice for volume calculation, (c) illustration of method of measuring the BRH, PRH, and RW, (d) CBCT at baseline, (e) fused CBCTs, (f) CBCT at 6 months postoperatively.

Baseline images were fused to 6‐month images using manual registration: initial manual alignment was performed, followed by automatic alignment through fixed landmarks on the jaw, such as the anterior nasal spine. As a result, we obtained a follow‐up image spatially aligned with the baseline scan, allowing the same measurement matrix to be applied and enhancing the precision of identifying the ridge center and ensuring consistency across all measurements [61, 62, 64, 65]. All measurements were first recorded on the primary image. Then the measurement on the primary image was left, and the primary image was concealed, leaving the secondary image. New measurements were subsequently recorded on the secondary image on the same plane direction and cut of the primary image, ensuring standardization. A bisecting vertical reference line corresponding to the long axis of the alveolar ridge was placed in the center of the extraction socket, and perpendicular horizontal reference lines were set at the most apical point of the extraction sites. Both mid BRH and PRH were measured parallel to the vertical reference line from the most coronal crest points, each to the horizontal reference line [17]. Buccopalatal RW was measured from the outermost surface of the buccal and palatal ridges of the extraction socket at 2 mm below the most coronal aspect of the bone crest [66]. After the superimposition, measurements were recorded. The loss in alveolar ridge dimensions was reported by subtracting final values from baseline values and expressed in millimeters and percentages (Figure 2).

#### 2.9.2 Patient‐Reported Outcome (Pain VAS Scores)

Self‐reported postoperative pain was reported by the patients every day for a week postoperatively using a 0–10 visual analogue scale (VAS) in which 0 means no pain, while 10 represents the worst pain possible [67].

#### 2.9.3 Implant Related Outcome

The need (yes/no) for additional bone augmentation for proper implant placement in the ideal 3D prosthetic position to allow for a minimum of 1‐mm circumferential bone support was assessed in the 6‐month CBCT scans [60]. This was done by virtual planning for implant placement with a virtual crown using Atomica AI software (version 8.0.1, Atlanta, USA, Figure 3a). Tapered implants were placed virtually in the selected sites of ARP. The diameter of the selected implants corresponded to the edentulous site. Thus, for edentulous spaces of central incisors, canines, and premolars, implants with a diameter of 4.3 mm were selected. For edentulous spaces of lateral incisors, implants with diameters of 3.2 mm were selected [68]. Mesiodistally, implants were positioned in the center of the edentulous span. Buccolingually, the buccal periphery of the implant was positioned at least 1 mm and no more than 2 mm palatal to the point of emergence of the adjacent teeth. Apically, the implant platform was positioned 3–4 mm apically to the cervical margin of the designed implant crown [69].

**FIGURE 3:**
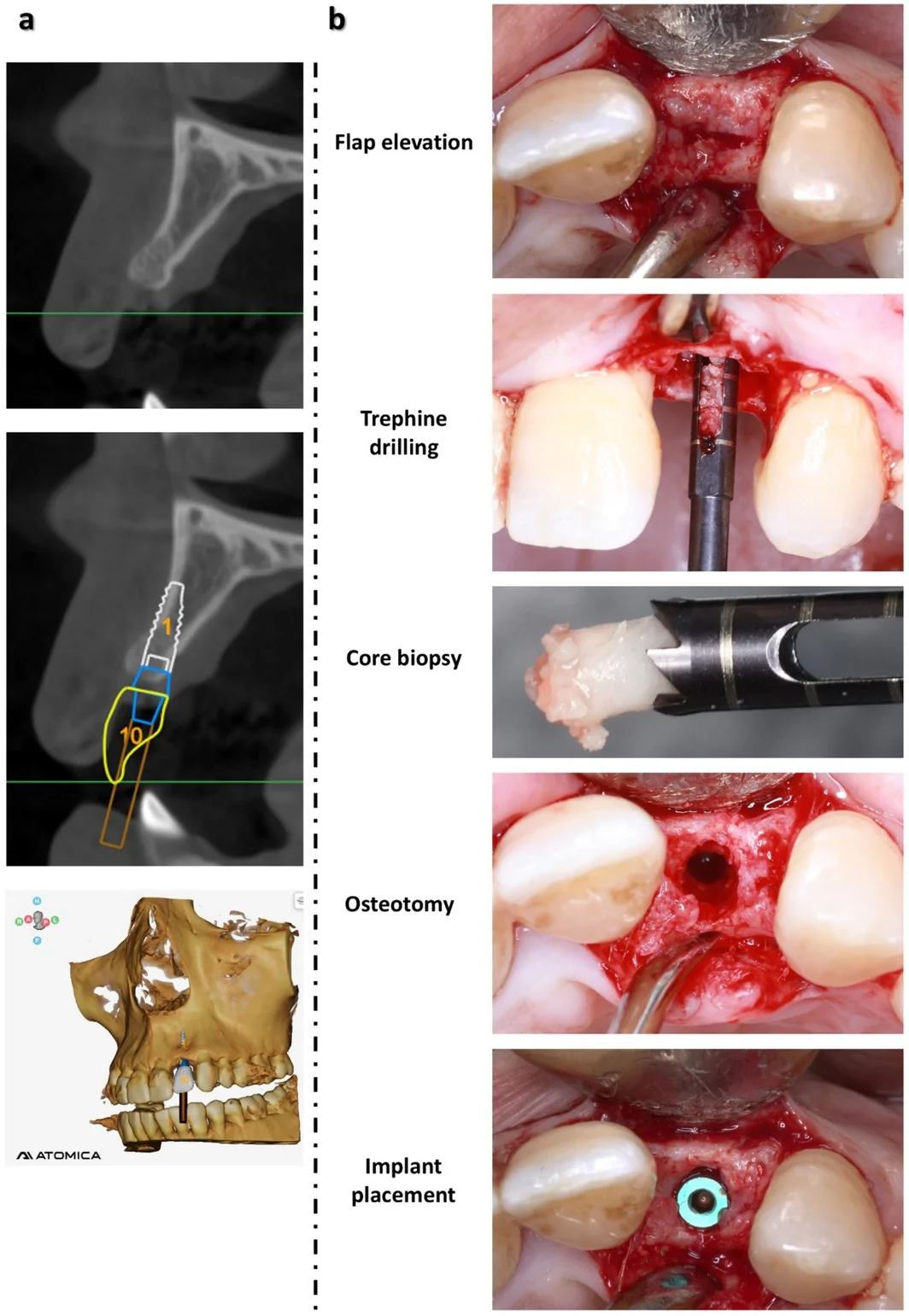
(a) CBCT at 6 months for virtual implant placement showing the need for additional bone augmentation. (b) Biopsy harvesting steps, using trephine drill, followed by dental implant placement.

#### 2.9.4 Histological Analysis

Microscopic examination and histomorphometric analysis were conducted on 18 core biopsies obtained from 18 patients during implant placement (six from each group). A trephine bur with an outer diameter of 3 mm and an inner diameter of 2 mm (THB30, Osung MND Co. Ltd., Gyeonggi‐do, South Korea, distributed by Osung USA, Irvine, California, USA) was used to obtain bone biopsies before implant placement (Figure 3b). The collected samples were preserved in 10% formalin solution, decalcified in 10% formic acid for a period of 10–15 days to be prepared for embedding in paraffin blocks, which were then sliced longitudinally into thin histological 5 μm sections. This was followed by staining the prepared histological sections with hematoxylin and eosin (H&E) stain to detect areas of newly formed bone and also to inspect any inflammatory changes. Also, Masson's trichromatic (MT) stain was used in other sections for histological inspection and histomorphometric analysis of the quality of the newly formed bone (Figure 4). A digital microscope (Leica Microsystems, Wetzlar, Germany) was used for the examination of all histologically stained sections, and photomicrographs of some prepared slides were captured. Photomicrographs were taken (Leica Microsystems, Heerbrugg, Switzerland) for capturing the histological sections examined under the microscope. Histomorphometric analysis for all the prepared MT‐stained sections was performed, using image analyzer software (Leica QWin 500 image analysis software, Leica Microsystems, Heerbrugg, Switzerland). Areas of newly formed bone, as unmineralized (osteoid) bone or mineralized (osseous) bone, along with the remaining graft particles and soft/fibrous tissue surrounding it were measured using a measuring frame with a surface area of 24 790 μm^2^. Five fields were carefully selected for each sample in each group and examined at a magnification of ×100. The mean percentages for each group were measured, calculated, and recorded [17].

**FIGURE 4:**
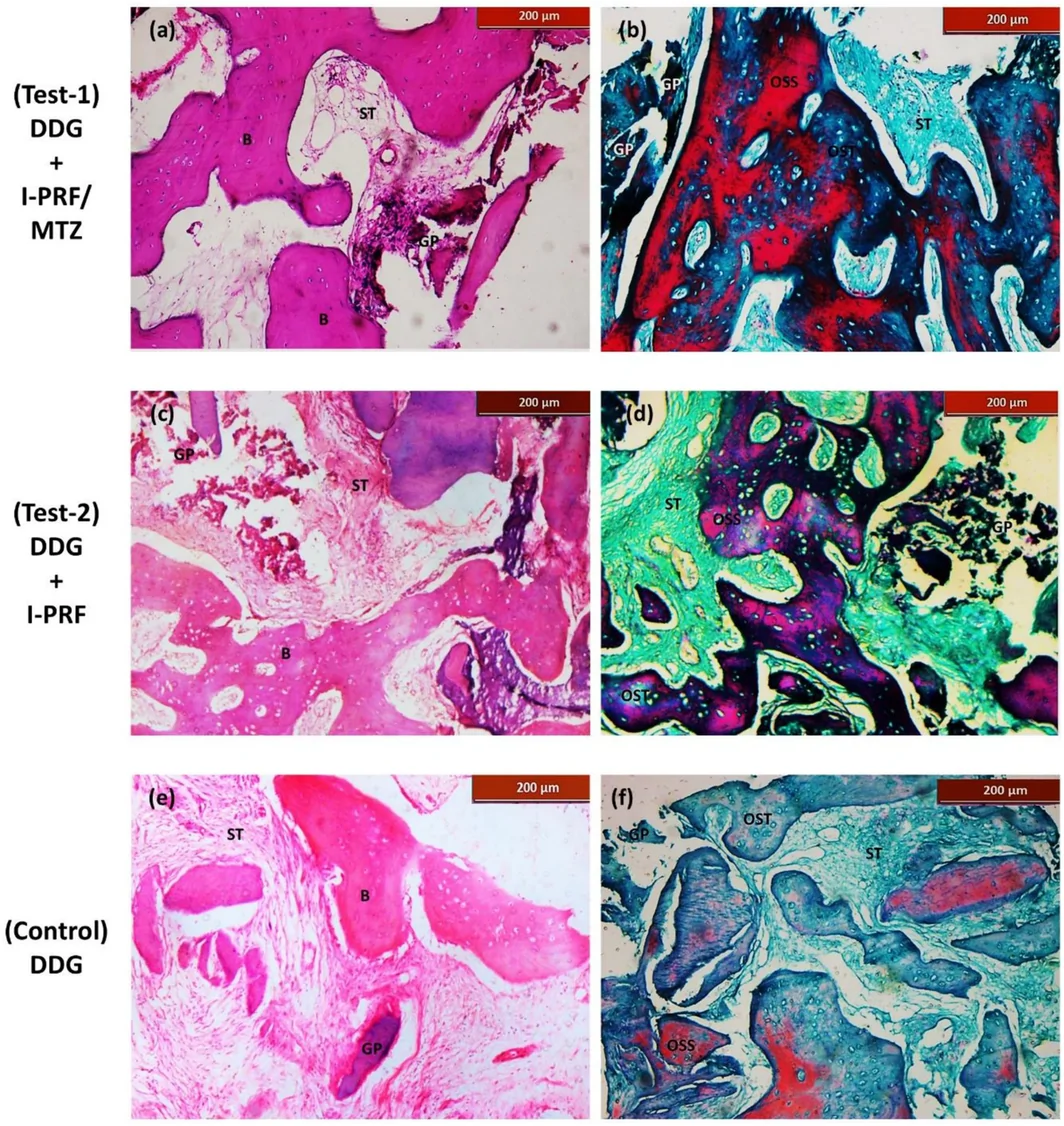
Qualitative histological examination. Representative microscopic images (100× magnification) with hematoxylin and eosin staining of histological sections of DDG + I‐PRF/MTZ (a), DDG + I‐PRF (c), and DDG (e) biopsies. Masson trichrome staining of histological sections of DDG + I‐PRF/MTZ (b), DDG + I‐PRF (d), and DDG (f) biopsies. B, newly formed bone; GP, graft particles; OSS, osseous tissue; OST, unmineralized osteoid tissue; ST, soft tissue.

### 2.10 Statistical Analysis

Data were explored for normality using tests of normality (Kolmogorov–Smirnov and Shapiro–Wilk tests). Age, ridge height, buccolingual ridge width, the socket volume, periapical lesion volume, and buccal bone thickness data showed normal (parametric) distribution, while pain scores data showed non‐normal (non‐parametric) distribution. Continuous data were presented as mean ± standard deviation (SD), and categorical data were presented as frequency and percentage. For parametric data, a paired *t*‐test was employed to test the changes by time within each group, and one‐way repeated measures ANOVA test was used to compare the three groups. In case ANOVA shows statistical significance, post hoc Tukey test with Bonferroni's adjustment was used for pairwise comparisons. For non‐parametric data, Kruskal‐Wallis test was used to compare the three groups. Additionally, a stepwise linear regression model was constructed, using BRH change after 6 months as the dependent variable, while study group, age, gender, tooth side, tooth type, socket volume, apical lesion volume, radiographic RW, and buccal bone thickness at baseline were the independent variables. The significance level was set at *p* ≤ 0.05. Statistical analysis was performed with IBM SPSS Statistics for Windows, Version 23.0. Armonk, NY: IBM Corp.

## 3 Results

Participants were recruited, treated, and followed up between May 2024 and June 2025. All participants in the three groups completed the follow‐up period and were included in the analysis (Figure S2). No unexpected adverse effects, such as swelling, edema, prolonged bleeding, infection, or loss of graft were observed, and complete soft tissue healing was evident in all groups at the end of the follow‐up time. The buccal bone at baseline was categorized as “thin” in 83.33%, 100%, and 94.44% of cases in DDG + I‐PRF/MTZ, DDG + I‐PRF, and DDG groups, respectively with no statistically significant difference between the groups (Chi‐Square, *p* = 0.151). There was no statistically significant difference between the groups at baseline regarding distribution of age and gender, tooth location, tooth type, extraction socket volume, or apical lesion volume (*p* > 0.05, Table 1).

**TABLE 1:** Descriptive statistics and results of two‐way repeated measures ANOVA test for comparisons between baseline characteristics in the three groups.

Baseline characteristics	DDG + I‐PRF/MTZ (*n* = 18)	DDG + I‐PRF (*n* = 18)	DDG (*n* = 18)	*p*
Age in years [mean ± SD]	31.56 ± 5.87	33.28 ± 6.39	32.94 ± 6.35	0.679
Gender [*n* (%)]				
Male	2 (11.1%)	1 (5.6%)	2 (11.1%)	0.802
Female	16 (88.9%)	17 (94.4%)	16 (88.9%)	
Tooth location [*n*, (%)]				
Right	13 (72.2%)	8 (44.4%)	6 (33.3%)	0.056
Left	5 (27.8%)	10 (55.6%)	12 (66.7%)	
Tooth type [*n*, (%)]				
Lateral incisor	11 (61.1%)	11 (61.1%)	8 (44.4%)	0.159
Canine	3 (16.7%)	6 (33.3%)	3 (16.7%)	
Premolar	4 (22.2%)	1 (5.6%)	7 (38.9%)	
Buccal bone thickness in mm [mean ± SD]	0.670 ± 0.25	0.629 ± 0.20	0.674 ± 0.22	0.799
Socket volume in mm^3^ [mean ± SD]	411.37 ± 98.77	362.62 ± 52.41	399.20 ± 95.41	0.210
Apical lesion volume in mm^3^ [mean ± SD]	101.73 ± 78.09	70.54 ± 50.38	61.42 ± 52.01	0.130

Abbreviations: DDG, demineralized dentin graft; I‐ PRF, injectable platelet‐ rich fibrin, MTZ, metronidazole.

### 3.1 Radiographic Analysis

The difference between groups concerning baseline dimensions was statistically insignificant (*p* > 0.05; ANOVA). A significant reduction in buccal and palatal ridge heights and buccolingual ridge width between baseline and 6 months of follow‐up was notable in all groups independently (*p* < 0.001, *t*‐test, Table 2).

**TABLE 2:** Mean (± SD) for buccal ridge height (BRH), palatal ridge height (PRH), average ridge height (ARH), ridge width (RW) (significant differences are marked with an asterisk; *p < 0.05).

Outcome	DDG + I‐PRF/MTZ (*n* = 18)	DDG + I‐PRF (*n* = 18)	DDG (*n* = 18)	Intergroup ANOVA
	Mean ± SD	Mean ± SD	Mean ± SD	*p*
Buccal ridge height (BRH)				
At baseline (mm)	11.35 ± 1.68	10.73 ± 1.21	10.35 ± 1.10	0.093
At 6 months (mm)	10.07 ± 1.66	9.25 ± 1.04	9.10 ± 0.96	0.055
Intra‐group *p*‐value	*p* < 0.001*	*p* < 0.001*	*p* < 0.001*	
Loss at 6 months (mm)	1.28 ± 0.51	1.47 ± 0.50	1.25 ± 0.50	0.361
Loss at 6 months (%)	11.38 ± 4.40	13.65 ± 3.87	11.94 ± 4.36	0.252
Palatal ridge height (PRH)				
At baseline (mm)	10.78 ± 2.13	10.14 ± 1.08	10.07 ± 0.99	0.300
At 6 months (mm)	9.25 ± 1.96	8.72 ± 1.00	8.60 ± 1.31	0.378
Intra‐group *p*‐value	*p* < 0.001*	*p* < 0.001*	*p* < 0.001*	
Loss at 6 months (mm)	1.53 ± 0.53	1.43 ± 0.47	1.47 ± 0.53	0.833
Loss at 6 months (%)	14.37 ± 4.70	14.04 ± 4.25	14.94 ± 6.26	0.868
Average ridge height (ARH)				
At baseline (mm)	11.06 ± 1.85	10.44 ± 1.10	10.21 ± 0.93	0.157
At 6 months (mm)	9.66 ± 1.75	8.99 ± 0.95	8.85 ± 0.99	0.141
Intra‐group *p*‐value	*p* < 0.001*	*p* < 0.001*	*p* < 0.001*	
Loss at 6 months (mm)	1.40 ± 0.45	1.45 ± 0.38	1.36 ± 0.36	0.786
Loss at 6 months (%)	12.83 ± 3.89	13.85 ± 3.08	13.39 ± 3.77	0.697
Ridge width (RW)				
At baseline (mm)	7.30 ± 0.91	7.12 ± 0.83	7.44 ± 1.30	0.648
At 6 months (mm)	6.03 ± 0.92	5.60 ± 0.44	6.00 ± 1.10	0.268
Intra‐group *p*‐value	*p* < 0.001*	*p* < 0.001*	*p* < 0.001*	
Loss at 6 months (mm)	1.28 ± 0.32	1.51 ± 0.93	1.44 ± 0.55	0.536
Loss at 6 months (%)	17.63 ± 4.64	20.33 ± 10.12	19.23 ± 6.26	0.547

Abbreviations: DDG, demineralized dentin graft; I‐ PRF, injectable platelet‐ rich fibrin, MTZ, metronidazole.

### 3.2 Pain VAS Scores

Pain VAS‐scores were significantly higher in the first 5 days after surgery in the DDG‐group (*p* < 0.05, Kruskal‐Wallis test, Table 3).

**TABLE 3:** Mean (± SD) for visual analogue scale (VAS) scores in the first 7 days postoperatively (significant differences are marked with an asterisk; *p < 0.05).

VAS scores	DDG + I‐PRF/MTZ (*n* = 18)	DDG + I‐PRF (*n* = 18)	DDG (*n* = 18)	Intergroup Kruskal‐Wallis
	Mean ± SD	Mean ± SD	Mean ± SD	*p*
VAS_1	2.39 ± 1.24	2.67 ± 0.84	3.94 ± 1.26	0.000*
VAS_2	1.28 ± 0.96	1.44 ± 0.78	3.61 ± 1.88	0.000*
VAS_3	0.89 ± 0.76	0.94 ± 0.87	3.17 ± 2.07	0.000*
VAS_4	0.22 ± 0.43	0.17 ± 0.38	1.33 ± 1.14	0.000*
VAS_5	0.00 ± 0.00	0.00 ± 0.00	0.39 ± 0.61	0.002*
VAS_6	0.00 ± 0.00	0.00 ± 0.00	0.11 ± 0.32	0.130
VAS_7	0.00 ± 0.00	0.00 ± 0.00	0.00 ± 0.00	NS

Abbreviations: DDG, demineralized dentin graft; I‐PRF, injectable platelet‐rich fibrin; MTZ, metronidazole; NS, not significant.

### 3.3 Implant Related Outcome

Additional bone augmentation at the time of implant placement was deemed necessary in two patients (11.11%) in each the DDG + I‐PRF/MTZ‐group and the DDG + I‐PRF‐group, while in four patients (22.22%) in the DDG group. This difference was statistically non‐significant (Chi‐Square, *p* > 0.05, Table S1).

### 3.4 Histological Evaluation and Histomorphometric Analyses

Regarding the H&E‐stained sections, areas of new bone formation without inflammatory changes were observed surrounding the residual graft particles in most of the specimens. On MT‐stained sections, soft unmineralized new (osteoid) bone, stained blue to green, while hard mineralized new (osseous) bone was colored red. In the DDG‐group, the histopathological examination of H&E‐stained sections revealed graft particles of variable size (showing dentinal tubules in large particles embedded in the soft fibrous stroma and surrounded by separated and disorganized newly formed bone trabeculae showing osteocytes in their lacunae and osteoblasts). The MT‐stained sections revealed that most of the newly formed bone was composed mainly of unmineralized osteoid bone interlaced with mineralized osseous bone. In the DDG + I‐PRF/MTZ‐group and the DDG + I‐PRF‐group variable size graft particles embedded in the soft fibrous stroma were surrounded by thick, interconnected, newly formed bone trabeculae (showing osteocytes in their lacunae). The MT‐stained sections illustrated that the newly formed bone in these groups was mainly composed of mineralized osseous bone interlaced with smaller areas of unmineralized osteoid bone color. In the DDG + I‐PRF/MTZ‐group, the mean percentage area of mineralized osseous tissue was 55.03% ± 3.65%, unmineralized osteoid tissue was 20.04% ± 5.20%, residual graft particles were 6.66% ± 3.84%, and soft tissue was 18.27% ± 4.48%. For the DDG + I‐PRF‐group, the mean percentage area of mineralized osseous tissue was 41.14% ± 7.01%, unmineralized osteoid tissue was 20.08% ± 5.67%, residual graft particles were 3.43% ± 1.12%, and soft tissue was 35.34% ± 11.76%. For the DDG‐group, the mean percentage area of mineralized osseous tissue was 30.21% ± 3.72%, unmineralized osteoid tissue was 30.26% ± 10.38%, residual graft particles were 3.42% ± 2.05%, and soft tissue was 36.11% ± 7.07%. The DDG + I‐PRF/MTZ‐group showed statistically higher mineralized bone formation than the DDG + I‐PRF‐group, which showed as well higher mineralized bone formation than the DDG‐group (*p* < 0.05, ANOVA, Table 3). One‐way ANOVA statistical test revealed statistical significance between the three groups regarding the unmineralized bone formation (*p* < 0.05). In addition, the DDG + I‐PRF/MTZ‐group demonstrated significantly less soft tissue formation than both the DDG + I‐PRF ‐group and DDG‐group (*p* < 0.05, ANOVA, Table 4).

**TABLE 4:** Histomorphometric analysis of percentages of histological tissue for each group.

Outcome	DDG + I‐PRF/MTZ	DDG + I‐PRF	DDG	ANOVA
	Mean ± SD	Mean ± SD	Mean ± SD	*p*
Mineralized osseous tissue %	55.03 ± 3.65^a^	41.14 ± 7.01^b^	30.21 ± 3.72^c^	0.000*
Unmineralized osteoid tissue %	20.04 ± 5.20^a^	20.08 ± 5.67^a^	30.26 ± 10.38^a^	0.048
Residual graft particles %	6.66 ± 3.84^a^	3.43 ± 1.12^a^	3.42 ± 2.05^a^	0.075
Soft tissue %	18.27 ± 4.48^a^	35.34 ± 11.76^b^	36.11 ± 7.07^b^	0.003*

*Note:* Means with different superscript letters are significant different (Bonferroni's post hoc test).

* The significance level is at the 0.05.

### 3.5 Stepwise Linear Regression Analysis

Stepwise linear regression analysis showed an inverse correlation between buccal bone thickness at baseline and BRH reduction (*p* < 0.05) as well as a direct correlation between RW at baseline and BRH reduction (*p* < 0.05, Table 5).

**TABLE 5:** Significant predictors of BRH loss according to stepwise linear regression analysis model.

	*β*	SE	95% CI		*p*
Group	0.193	0.087	−0.057	0.293	0.180
Gender	0.115	0.244	−0.295	0.690	0.424
Age	0.097	0.011	−0.014	0.030	0.469
Tooth location	−0.018	0.146	−0.312	0.275	0.902
Tooth type	−0.395	0.146	−0.535	0.052	0.105
Socket volume	0.195	0.001	−0.001	0.004	0.382
Apical lesion volume	0.166	0.001	−0.001	0.004	0.265
Buccal bone thickness	−0.295	0.333	−1.351	−0.009	0.047*
Ridge width (RW) at baseline	0.382	0.086	0.016	0.361	0.033*

*Note:* Significant differences are marked with asterisk; *p < 0.05.

Abbreviations: *β*, regression coefficient; CI, confidence interval; SE standard error.

## 4 Discussion

The maxillary esthetic zone is particularly susceptible to bone resorption following tooth extraction due to the predominance of a thin labial bone plate [2, 70]. Although various ARPs have been proposed as effective strategies to mitigate post‐extraction bone loss in such compromised sites [21, 71, 72, 73, 74, 75], none have completely prevented ridge resorption [76], and no single approach has consistently outperformed others [70]. In this context, a combination of different forms of PRF with mineralized or demineralized autogenous dentin grafts was suggested for further improvement in bone structure and dimensions in post‐extraction ARP of intact and periodontally infected sockets [15, 21, 41]. The aim of the present pilot randomized controlled trial was to compare for the first time the DDG + I‐PRF/MTZ, DDG + I‐PRF and DDG alone in ARP of extraction sockets with periapical pathological lesions in the maxillary esthetic region.

In the current investigations, all groups showed similar effectiveness in ARP, with no significant differences observed between DDG + I‐PRF/MTZ, DDG + I‐PRF, and DDG alone regarding changes in BRH. The results were comparable to previous studies investigating the effect of DDG and DDG + I‐PRF on ARP [17, 41]. The regression analysis demonstrated that irrespective of the treatment group, buccal bone thickness and ridge width at baseline were significant predictors of the reduction of BRH after 6 months of ARP. In accordance with previous studies [77, 78], the thicker the buccal bone plate the lesser the reduction in BRH after 6 months within the scope of the three ARP protocols investigated. On the contrary, the larger the RW at baseline, the higher the reduction in buccal ridge height, which might be attributed to the instability of the blood clot at wider socket orifices. This could be further correlated to the socket dimensions, as wide RW would be linked to a greater volume, which could affect bone healing and regeneration, similar to the effect of a critical defect size on bone regeneration [79, 80]. These findings further align with the established biomechanics and biological principles of socket remodeling. Sockets with thin buccal plates (≤ 1 mm), which were dominant in participants of the current study, are well known to undergo substantially more resorption following extraction. This is attributed to the predominance of bundle bone, a tooth‐dependent bone that rapidly resorbs once the periodontal ligament is lost [2, 81, 82]. Thin cortical plates further have reduced vascularity and diminished structural stability, making them more susceptible to collapse during the early remodeling phase [83]. This biological vulnerability explains the inverse correlation observed: the thinner the buccal plate at baseline, the more pronounced the vertical loss after healing. Similar associations have been reported in morphometric and CBCT‐based analyses, showing that wider ridges tend to experience proportionally greater volumetric remodeling [77, 84]. These relationships emphasize that baseline morphology strongly determines the extent of ridge dimensional changes, even when a graft material is used.

All groups further showed no significant differences between DDG + I‐PRF/MTZ, DDG + I‐PRF, and DDG alone regarding PRH and RW as well. The present results on the effectiveness of all three types of grafts in ARP are in accordance with earlier studies investigating the effect of the demineralization processes on autogenous dentin graft in ARP procedures [17], or comparing the effect of autogenous DDG mixed with I‐PRF to DDG alone [41], which similarly demonstrated that all grafts were comparably effective with no statistically significant differences noted. Although the reduction in BRH and PRH in the present study appears to be slightly higher than the aforementioned studies, the extraction sockets of the present study were all chronically infected with apical lesions, which may increase and accelerate bone resorption of the surrounding socket walls [5]. Implant placement feasibility was 100% in all groups, but it was determined that approximately 22.22% of the sites in the DDG group would require additional bone augmentation at the time of implant placement, as compared with sites in DDG + I‐PRF/MTZ and DDG + I‐PRF groups (11.11% each). These findings align with a recent systematic review, which reported that ridge augmentation was required either before or during implant placement in approximately 12.5% to 13.9% of cases after ARP [85].

Histological evaluations provide an insight for detecting the quality of newly formed bone (either soft/unmineralized or hard/mineralized) as well as early immune responses surrounding graft particles prior to the appearance of clinical or radiographic evidence [86, 87]. Qualitatively, similar to earlier findings, histological sections of autogenous dentin graft mixed with I‐PRF [20, 41, 88] and DDG [17], groups demonstrated new bone formation with no obvious inflammatory infiltrates, with the highest bone mineralization in the DDG + I‐PRF/MTZ‐group, followed by the DDG + I‐PRF‐group. The significant difference in mineralized bone formation in both groups might be due to the additional antibacterial synergistic effect of metronidazole in the chronically‐infected sockets, thus reducing the effect of infection on bone resorption more rapidly in the healing sites [39, 89]. Moreover, the presence of I‐PRF in both groups enhanced both mineralized and unmineralized bone formation, as I‐PRF would promote the osteogenic differentiation even under an inflammatory environment, indicating that the anti‐inflammatory effect and regenerative potential of I‐PRF can counterbalance the negative inflammatory impact induced by chronic infection [90, 91]. Similarly, in an investigation by Kumar et al. [92], PRF with 2% metronidazole showed enhanced and faster bone healing following surgical extraction of impacted third molars. In all groups, the DDG particles seemed to be resorbed and encircled by osteoid tissues with vascularized bone marrow spaces, suggesting new bone formation [17].

In the present investigations, smokers were excluded to avoid adverse effects on bone healing and increased post‐extraction alveolar crest loss due to interferences with the formation of the fibrin mesh, reduced blood supply, and cell viability, and consequently detrimental effects on bone quality and regenerative capacity [93, 94]. Exclusion of molars was done since bone quality, as well as the size and configuration of molar sockets is different from non‐molar teeth with dissimilar healing time and events [5, 70]. Moreover, multiple teeth indicated for extraction or teeth with adjacent periodontally compromised teeth were further excluded, as this might influence the extent of the vertical and horizontal oral tissue reduction [2, 3]. Radiographic and histomorphometric analyses were carried out at 6 months of ARP to allow optimum bone fill, and because the dimensional changes are more distinct between 3 and 6 months of healing [5, 95, 96]. Baseline CBCT scans were obtained on the same day after extraction and ARP [41, 65, 97] to ensure measurement accuracy, as even atraumatic extraction may induce minimal alterations of socket morphology, such as slight expansion or chipping of the thin buccal plate. However, this timing of baseline CBCT imaging could be a potential limitation as other studies recommended pre‐extraction imaging to detect any defects, to evaluate native alveolar anatomy before any surgical manipulation, and to provide an accurate preoperative diagnosis [75, 98].

DDG is less costly in comparison with other bone substitutes and appears to exhibit low or even negligible complication rates [20]. The demineralization process exposes growth factors such as IGF‐II, BMP‐2, and TGF‐β, which promote osteogenic differentiation and bone formation [21, 41]. Additionally, the combination of I‐PRF with dentin particles enriches them with growth factors [99], in addition to enhancing their handling characteristics forming a “sticky graft” [41, 100]. However, the preparation of I‐PRF requires additional venipuncture, blood processing, and chairside time, representing additional biologic and procedural load and cost in comparison to unassisted socket healing [70]. Consequently, its use as an adjunct during ARP should be considered on a case‐by‐case basis [40, 101]. I‐PRF could provide a liquid and easily usable platelet concentration for the incorporation of a range of sustained local release of biomaterials, like antibiotics [24, 27, 28, 29, 40, 102], with adjunctive locally delivered antibiotics during surgical periodontal or implant therapy demonstrating an enhancement in clinical parameters for up to 6 months postoperatively [40, 103]. Yet, this approach must be balanced with the broader public‐health concern of antimicrobial resistance. Therefore, the use of local antibiotics should be limited to clearly indicated clinical scenarios where the potential benefits outweigh the risk [104].

Still, the current trial's result should be cautiously interpreted in the light of its limitations. A notable limitation of this study is the absence of a negative control group consisting of unassisted socket healing. Including such a group would have allowed a direct comparison between grafted and non‐grafted infected sockets and would have helped isolate the true additive effect of the tested interventions beyond the natural healing baseline. Moreover, although dentin particles were prepared chairside using a bone mill, standardization of DDG particle size was not feasible, which may have introduced variability in graft behavior. Another limitation is the absence of quantitative soft tissue assessments, given the known stimulatory effects of I‐PRF on soft tissue regeneration and wound healing [40]. It is important to distinguish that soft tissue remodeling does not inevitably reflect hard tissue changes following ARP, as improvements in bone healing may happen independently of soft tissue outcomes [105]. Additional limitations include the limited sample size, the reduced number of biopsies available for histomorphometric analysis, and the lack of evaluation of chairside preparation time and cost across the examined treatment modalities. Furthermore, analgesic consumption was not recorded, limiting insight into postoperative discomfort beyond VAS scoring. Finally, this trial was prospectively registered using anticipated estimates for sample size and study timelines. Following study completion, the registration was updated to reflect the actual recruitment dates and participant numbers, without any changes to the trial design, primary or secondary outcomes, or intervention procedures.

## 5 Conclusions

All three grafting protocols were similarly effective for ARP in chronically infected sockets. The addition of I‐PRF with MTZ promoted the newly formed bone quality with higher mineralization.

## Author Contributions

**Mohamed Talaat Elbehwashy:** conceptualization, methodology, writing, review and editing, and investigation. **Manal Mohamed Hosny:** supervision, project administration, writing, review, and editing. **Ahmed Elfana:** formal analysis, writing – review and editing, data curation. **Heba Ahmed Saleh:** formal analysis, data curation, writing – review and editing. **Karim Fawzy El‐Sayed:** conceptualization, formal analysis, writing – original draft, writing – review and editing, validation.

## Funding

The authors received no funding for this work.

## Disclosure

This manuscript did not use artificial intelligence in any capacity.

## Ethics Statement

All procedures performed in studies involving human participants were in accordance with the ethical standards of the institutional and/or national research committee and with the 2024 Helsinki Declaration and its later amendments or comparable ethical standards. Research protocol, informed consent templates, and biological sample collection requests were approved by the Research Ethics Committee, Faculty of Dentistry, Cairo University, in September 2022 (IRB number: 22|09|10).

## Consent

All participants included in the study gave written informed consent before their participation.

## Conflicts of Interest

The authors declare no conflicts of interest.

## Supporting information


**Table S1:** Comparison of need for augmentation for implant placement between the study groups.
**Figure S1:** Periapical radiographs taken preoperatively.
**Figure S2:** Flow diagram of patients' recruitment.

## Data Availability

The data that support the findings of this study are available from the corresponding author upon reasonable request.
