# Supplementary material for: Autogenous Demineralized Dentin Graft–Based Strategies With or Without I‐PRF and Metronidazole for Ridge Preservation in Infected Sockets: A Randomized Clinical Trial
Source: J Periodontal Res. 2026 Feb 20;61(9):1033–48. doi: 10.1111/jre.70092 (PMC13768985; doi:10.1111/jre.70092)
Supplement: Supplementary file 1 [file JRE-61-1033-s001.docx]

**Table S1:** Comparison of need for augmentation for implant placement between the study groups

| **Need for augmentation** | **DDG + I-PRF + MTZ (Test-1 n=18)** | **DDG + I-PRF (Test-2 n=18)** | **DDG**  **(Control n=18)** | **p-value** |
| --- | --- | --- | --- | --- |
| No [n(%)] | 16 (88.89%) | 16 (88.89%) | 14 (77.78%) | 0.556 |
| Yes [n(%)] | 2 (11.11%) | 2 (11.11%) | 4 (22.22%) |  |

**Abbreviations:** DDG, demineralized dentin graft; I- PRF, injectable platelet- rich fibrin, MTZ, metronidazole.

**Figure S1:** Periapical radiographs taken preoperatively

**CONSORT 2010 Flow Diagram**

## Enrollment

Randomized (n= 54)

Excluded (n= 12)

♦  Not meeting inclusion criteria (n= 9)

♦  Declined to participate (n= 3)

♦  Other reasons (n= 0)

Assessed for eligibility (n= 66)

## Allocation

Analysed (n= 18)
♦ Excluded from analysis (give reasons) (n= 0)

## Analysis

Analysed (n= 18)
♦ Excluded from analysis (give reasons) (n= 0)

Analysed (n= 18)
♦ Excluded from analysis (give reasons) (n= 0)

Lost to follow-up (give reasons) (n= 0)

Discontinued intervention (give reasons) (n= 0)

Lost to follow-up (give reasons) (n= 0)

Discontinued intervention (give reasons) (n= 0)

Lost to follow-up (give reasons) (n= 0)

Discontinued intervention (give reasons) (n= 0)

## Follow-Up

Allocated to intervention (n=18)

♦ Received allocated intervention (n= 18)

♦ Did not receive allocated intervention (give reasons) (n= 0)

Allocated to intervention (n= 18)

♦ Received allocated intervention (n= 18)

♦ Did not receive allocated intervention (give reasons) (n= 0)

Allocated to intervention (n= 18)

♦ Received allocated intervention (n= 18)

♦ Did not receive allocated intervention (give reasons) (n= 0)

**Figure S2:** Flow diagram of patients’ recruitment
